# Supplementary material for: Histidine transport is essential for the growth of Staphylococcus aureus at low pH
Source: PLoS Pathog. 2024 Jan 16;20(1):e1011927. doi: 10.1371/journal.ppat.1011927 (PMC10817146; doi:10.1371/journal.ppat.1011927)
Supplement: S5 Table — (DOCX) [file ppat.1011927.s005.docx]

**S5 Table:** Bacterial strains used in this study

| **Unique ID** | **Strain name and resistance** | **Reference** |
| --- | --- | --- |
|  | ***E. coli* strains** |  |
| ANG243 | XL1-Blue pCL55; Amp 100 \|µg/ml | (1) |
| ANG1141 | CLG190 | Dana Boyd; (2) |
| ANG6028 | CLG190 pCL55-*0846*; Amp 100 µg/ml | This study |
|  | ***S. aureus* strains** |  |
| ANG3729 | TM283 (USA300-TCH1516 without pUSA300HOUMR) | (3, 4) |
| ANG2624 | JE2 (WT) | (5) |
| ANG4019 | JE2 *graS::Tn* (NE1756); Erm 10 µg/ml | (5) |
| ANG4008 | JE2 *vraG::Tn* (NE70); Erm 10 µg/ml | (5) |
| ANG4223 | Strain 923 (WT) | (6) |
| ANG4226 | Strain 923 Δ*vraS*; Cam 5 µg/ml | (6) |
| ANG4227 | Strain 923 Δ*vraR*; Cam 5 µg/ml | (6) |
| ANG6295 | JE2 *mprF::Tn* (NE1360); Erm 10 µg/ml | (5) |
| ANG5968 | JE2 *fmtA::Tn* (NE1022); Erm 10 µg/ml | (5) |
| ANG1575 | LAC* (WT) | (7) |
| ANG2395 | LAC* Δ*dltD* | (8) |
| ANG3969 | JE2 *SAUSA300_0482::Tn* (NE251); Erm 10 µg/ml | (5) |
| ANG3911 | JE2 *SAUSA300_0957::Tn* (NE1384); Erm 10 µg/ml | (5) |
| ANG5979 | JE2 *sagB::Tn* (NE1909); Erm 10 µg/ml | (5) |
| ANG3909 | JE2 *spdC::Tn* (NE1099); Erm 10 µg/ml | (5) |
| ANG5969 | JE2 *srrA::Tn* (NE1309); Erm 10 µg/ml | (5) |
| ANG3941 | JE2 *qoxB::Tn* (NE732); Erm 10 µg/ml | (5) |
| ANG5957 | JE2 *qoxA::Tn* (NE92); Erm 10 µg/ml | (5) |
| ANG5967 | JE2 *SAUSA300_0846::Tn* (NE967); Erm 10 µg/ml | (5) |
| ANG6197 | JE2 *SAUSA300_0846::Tn* transduced; Erm 10 µg/ml | This study |
| ANG5970 | JE2 *SAUSA300_2389::Tn* (NE1400); Erm 10 µg/ml | (5) |
| ANG5963 | JE2 *SAUSA300_0429::Tn* (NE620); Erm 10 µg/ml | (5) |
| ANG5964 | JE2 *SAUSA300_0543::Tn* (NE802); Erm 10 µg/ml | (5) |
| ANG5959 | JE2 *SAUSA300_0481::Tn* (NE188); Erm 10 µg/ml | (5) |
| ANG5972 | JE2 *SAUSA300_1518::Tn* (NE1474); Erm 10 µg/ml | (5) |
| ANG5955 | JE2 *SAUSA300_1636::Tn* (NE22); Erm 10 µg/ml | (5) |
| ANG5966 | JE2 *lepA::Tn* (NE865); Erm 10 µg/ml | (5) |
| ANG5978 | JE2 *SAUSA300_0759::Tn* (NE1891); Erm 10 µg/ml | (5) |
| ANG2631 | JE2 *noc::Tn* (NE486); Erm 10 µg/ml | (5) |
| ANG4016 | JE2 *SAUSA300_2055::Tn* (NE1495); Erm 10 µg/ml | (5) |
| ANG5971 | JE2 *SAUSA300_1043::Tn* (NE1462); Erm 10 µg/ml | (5) |
| ANG5608 | JE2 *lytH::Tn* (NE1369); Erm 10 µg/ml | (5) |
| ANG6129 | JE2 *codY::Tn* (NE1555); Erm 10 µg/ml | (5) |
| ANG6049 | LAC* *SAUSA300_0846::Tn*; Erm 10 µg/ml | This study |
| ANG6293 | LAC* *codY::Tn*; Erm 10 µg/ml | This study |
| ANG113 | RN4220 (WT) | (9) |
| ANG266 | RN4220 pCL55; Cam 7.5 µg/ml | (10) |
| ANG6069 | RN4220 pCL55-*0846;* Cam 7.5 µg/ml | This study |
| ANG3795 | LAC* pCL55, Cam 7.5 µg/ml | (11) |
| ANG6078 | LAC* *SAUSA300_0846::Tn* pCL55; Erm 10 µg/ml, Cam 7.5 µg/ml | This study |
| ANG6076 | LAC* *SAUSA300_0846::Tn* pCL55-*0846*; Erm 10 µg/ml, Cam 7.5 µg/m | This study |
| ANG6039 | JE2 *cpa1-1::Tn* (NE1504); Erm 10 µg/ml | (5) |
| ANG6040 | JE2 *cpa1-2::Tn* (NE366); Erm 10 µg/ml | (5) |
| ANG4556 | JE2 *cpa2::Tn* (NE308); Erm 10 µg/ml | (5) |
| ANG6037 | JE2 *nhaC1::Tn* (NE1470); Erm 10 µg/ml | (5) |
| ANG6038 | JE2 *nhaC2::Tn* (NE1214); Erm 10 µg/ml | (5) |
| ANG6080 | LAC* *SAUSA300_0846::Tn* *S-1;* Erm 10 µg/ml | This study |
| ANG6085 | LAC* *SAUSA300_0846::Tn* *S-2;* Erm 10 µg/ml | This study |
| ANG6087 | LAC* *SAUSA300_0846::Tn* *S-3;* Erm 10 µg/ml | This study |
| ANG6090 | LAC* *SAUSA300_0846::Tn* *S-4;* Erm 10 µg/ml | This study |
| ANG6099 | LAC* *SAUSA300_0846::Tn* *S-5;* Erm 10 µg/ml | This study |
| ANG6111 | LAC* *SAUSA300_0846::Tn* *S-6;* Erm 10 µg/ml | This study |
| ANG6113 | LAC* *SAUSA300_0846::Tn* *S-7;* Erm 10 µg/ml | This study |
| ANG6118 | LAC* *SAUSA300_0846::Tn* *S-8;* Erm 10 µg/ml | This study |
| ANG6126 | JE2 *hutU::Tn* (NE435)*;* Erm 10 µg/ml | (5) |
| ANG6127 | JE2 *hutH::Tn* (NE570)*;* Erm 10 µg/ml | (5) |

**References**

1. Lee CY, Buranen SL, Ye ZH. Construction of single-copy integration vectors for *Staphylococcus aureus*. Gene. 1991;103(1):101-5.

2. Gründling A, Gonzalez MD, Higgins DE. Requirement of the *Listeria monocytogenes* broad-range phospholipase PC-PLC during infection of human epithelial cells. J Bacteriol. 2003;185(21):6295-307.

3. Coe KA, Lee W, Stone MC, Komazin-Meredith G, Meredith TC, Grad YH, et al. Multi-strain Tn-Seq reveals common daptomycin resistance determinants in *Staphylococcus aureus*. PLoS Pathog. 2019;15(11):e1007862.

4. Santiago M, Matano LM, Moussa SH, Gilmore MS, Walker S, Meredith TC. A new platform for ultra-high density *Staphylococcus aureus* transposon libraries. BMC Genomics. 2015;16(1):252.

5. Fey PD, Endres JL, Yajjala VK, Widhelm TJ, Boissy RJ, Bose JL, et al. A genetic resource for rapid and comprehensive phenotype screening of nonessential *Staphylococcus aureus* genes. mBio. 2013;4(1):e00537-12.

6. Boyle-Vavra S, Yin S, Jo DS, Montgomery CP, Daum RS. VraT/YvqF is required for methicillin resistance and activation of the VraSR regulon in *Staphylococcus aureus*. Antimicrob Agents Chemother. 2013;57(1):83-95.

7. Boles BR, Thoendel M, Roth AJ, Horswill AR. Identification of genes involved in polysaccharide-independent *Staphylococcus aureus* biofilm formation. PLoS One. 2010;5(4):e10146.

8. Ledger EVK, Mesnage S, Edwards AM. Human serum triggers antibiotic tolerance in *Staphylococcus aureus*. Nat Commun. 2022;13(1):2041.

9. Kreiswirth BN, Lofdahl S, Betley MJ, O'Reilly M, Schlievert PM, Bergdoll MS, et al. The toxic shock syndrome exotoxin structural gene is not detectably transmitted by a prophage. Nature. 1983;305(5936):709-12.

10. Corrigan RM, Campeotto I, Jeganathan T, Roelofs KG, Lee VT, Gründling A. Systematic identification of conserved bacterial c-di-AMP receptor proteins. Proc Natl Acad Sci U S A. 2013;110(22):9084-9.

11. Schuster CF, Bellows LE, Tosi T, Campeotto I, Corrigan RM, Freemont P, et al. The second messenger c-di-AMP inhibits the osmolyte uptake system OpuC in *Staphylococcus aureus*. Sci Signal. 2016;9(441):ra81.
