# Supplementary material for: Histidine transport is essential for the growth of Staphylococcus aureus at low pH
Source: PLoS Pathog. 2024 Jan 16;20(1):e1011927. doi: 10.1371/journal.ppat.1011927 (PMC10817146; doi:10.1371/journal.ppat.1011927)
Supplement: S6 Table — (DOCX) [file ppat.1011927.s006.docx]

**S6 Table.** qRT-PCR primers and probes used in this study

| **Gene** | **Primers** | **Probe** |
| --- | --- | --- |
| *0846* | GTTTTGGTTTATGTTTCCCGTATGTGT | TTTGCTGGAAAATTTG |
|  | AATTGGGTGATTTGCCTTTGCA |  |
| *hisD* | GGCACAGAAACGATACCTAAAGTTG | CCAGGCAATCAATTTG |
|  | ACCTACTTGTCCAAATAAATATTTCTTGGCA |  |
| *hisG* | GCGAAACCTGAAACGACCAATTAT | TCGTTGCGATTTTACG |
|  | CCTTTTGATTTAAAATATGTTTCAGCAGTATGAACA |  |
| *gyrB* | CGCACGTACAGTGGTTGAAAA | TATTATGGCGGCACGT |
|  | CGTGTTACTTCACGCGCTTTT |  |
