# Supplementary material for: Histidine transport is essential for the growth of Staphylococcus aureus at low pH
Source: PLoS Pathog. 2024 Jan 16;20(1):e1011927. doi: 10.1371/journal.ppat.1011927 (PMC10817146; doi:10.1371/journal.ppat.1011927)
Supplement: S1 Fig — Circular plots for Tn-libraries A and B with the two outer rings depicting genes located on the (+) (blue) or (−) (green) strand in S. aureus strain. The inner three rings show the histograms of transposon insertions on a per gene basis after growth of the libraries in TSB pH 7.3 (red), pH 5.5 (purple), or pH 4.5 (blue) for 10 generations. (DOCX) [file ppat.1011927.s007.docx]

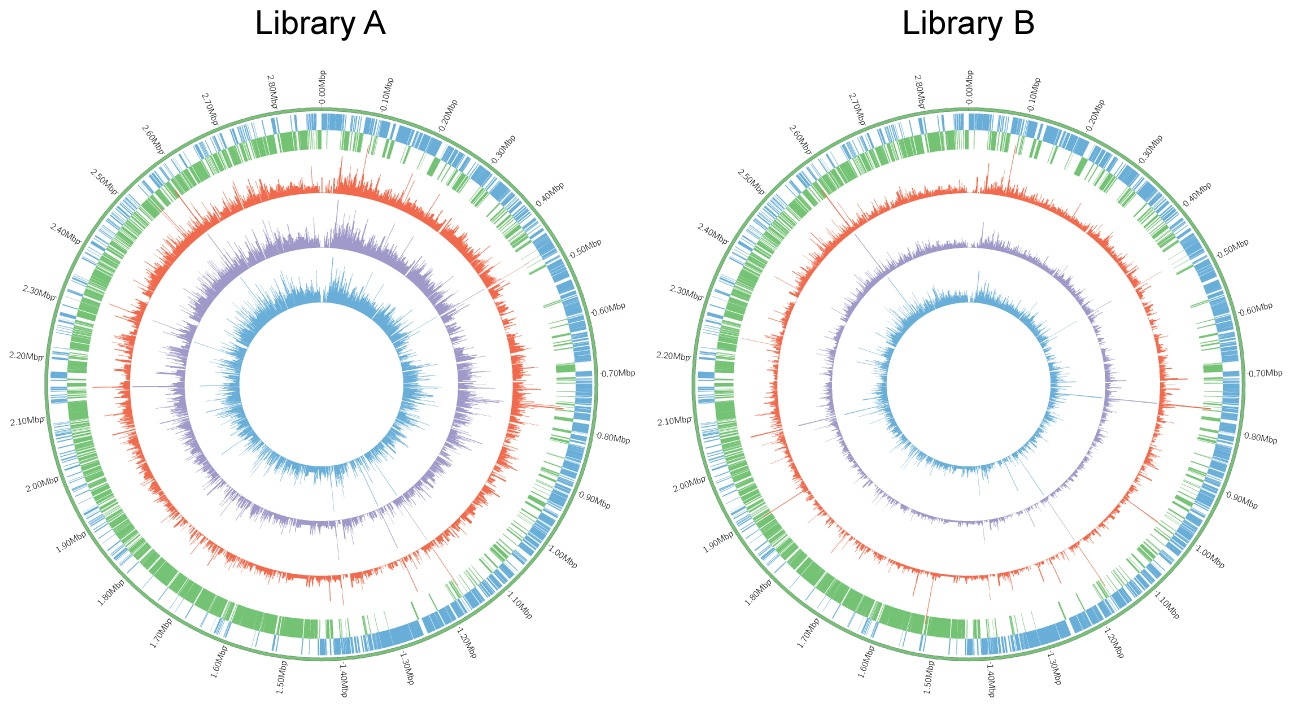


**S1 Fig: Circular plots showing the transposon insertion density along the *S. aureus* genome at different pH growth conditions**. Circular plots for Tn-libraries A and B with the two outer rings depicting genes located on the (+) (blue) or (−) (green) strand in *S. aureus* strain. The inner three rings show the histograms of transposon insertions on a per gene basis after growth of the libraries in TSB pH 7.3 (red), pH 5.5 (purple), or pH 4.5 (blue) for 10 generations.
