# Supplementary material for: Histidine transport is essential for the growth of Staphylococcus aureus at low pH
Source: PLoS Pathog. 2024 Jan 16;20(1):e1011927. doi: 10.1371/journal.ppat.1011927 (PMC10817146; doi:10.1371/journal.ppat.1011927)
Supplement: S2 Fig — (A-H) Bacterial growth on TSA pH 7.3 plates. Overnight cultures of the indicated WT and mutant strains were serially diluted and spotted on TSA pH 7.3 plates. Images were taken following 24 h incubation at 37°C. Each image is a representative of three experiments. (DOCX) [file ppat.1011927.s008.docx]

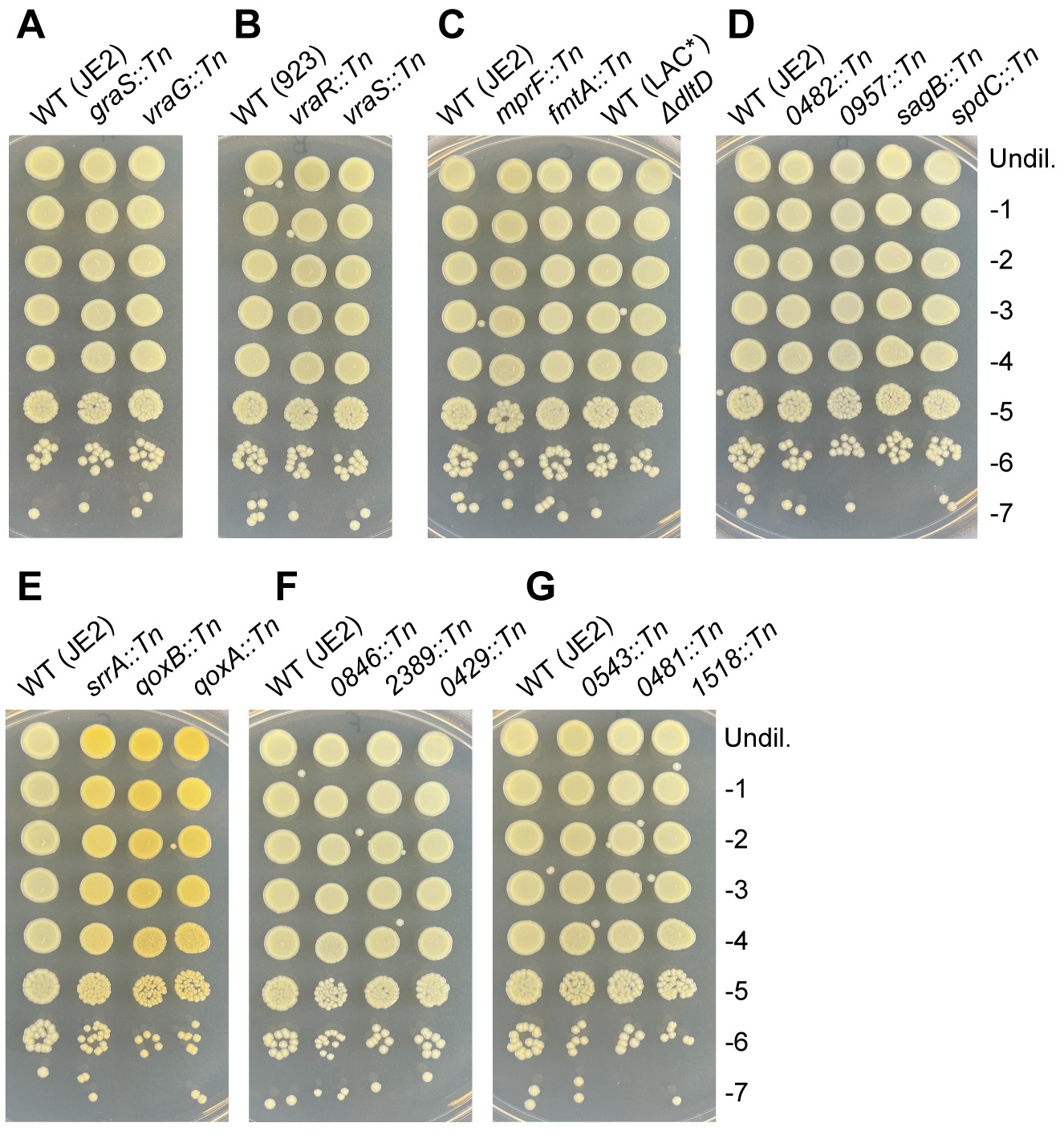


**S2 Fig: Growth plate analysis of *S. aureus* mutant strains with transposon insertions in genes identified as essential for growth at pH 4.5.** (A-H) Bacterial growth on TSA pH 7.3 plates. Overnight cultures of the indicated WT and mutant strains were serially diluted and spotted on TSA pH 7.3 plates. Images were taken following 24 h incubation at 37 °C. Each image is a representative of three experiments.
