# Supplementary material for: Histidine transport is essential for the growth of Staphylococcus aureus at low pH
Source: PLoS Pathog. 2024 Jan 16;20(1):e1011927. doi: 10.1371/journal.ppat.1011927 (PMC10817146; doi:10.1371/journal.ppat.1011927)
Supplement: S3 Fig — (A-D) Bacterial growth on TSA plates. Overnight cultures of the indicated WT and mutant strains were serially diluted and spotted on (A-B) TSA pH 7.3 plates or (C-D) TSA pH 4.5 plates. Images were taken following 24 h incubation at 37°C. Each image is a representative of three experiments. (DOCX) [file ppat.1011927.s009.docx]

**
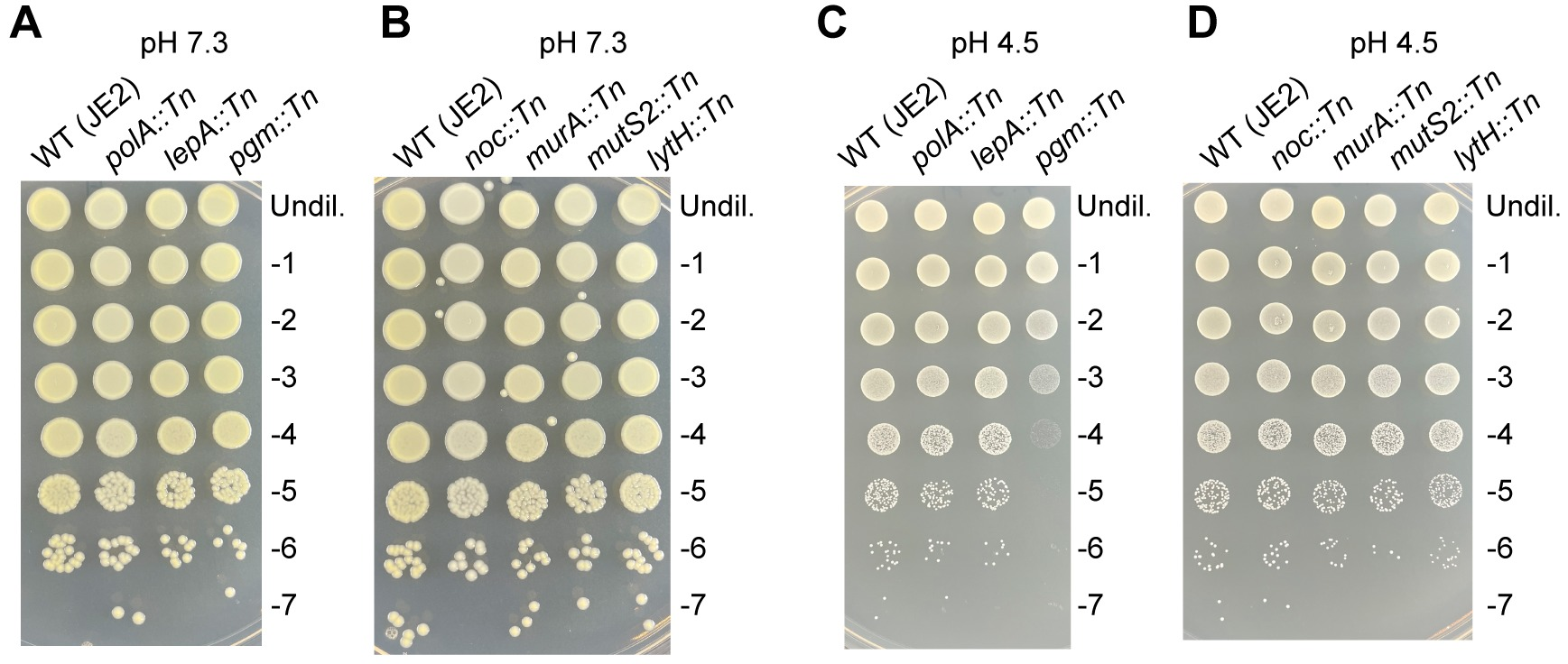
**

**S3 Fig: Growth plate analysis of *S. aureus* mutant strains with transposon insertions in genes identified as detrimental for growth at pH 4.5.** (A-D) Bacterial growth on TSA plates. Overnight cultures of the indicated WT and mutant strains were serially diluted and spotted on (A-B) TSA pH 7.3 plates or (C-D) TSA pH 4.5 plates. Images were taken following 24 h incubation at 37 °C. Each image is a representative of three experiments.
