# Supplementary material for: Histidine transport is essential for the growth of Staphylococcus aureus at low pH
Source: PLoS Pathog. 2024 Jan 16;20(1):e1011927. doi: 10.1371/journal.ppat.1011927 (PMC10817146; doi:10.1371/journal.ppat.1011927)
Supplement: S4 Fig — WT S. aureus LAC* containing the empty pCL55 (WT EV) was grown in CDM pH 4.3 medium with decreasing concentrations of histidine ranging from 130 μM to 0.2 μM or in the absence of histidine as indicated by the different symbols in the figure legend. The average OD600 readings from three experiments were plotted. (DOCX) [file ppat.1011927.s010.docx]

**
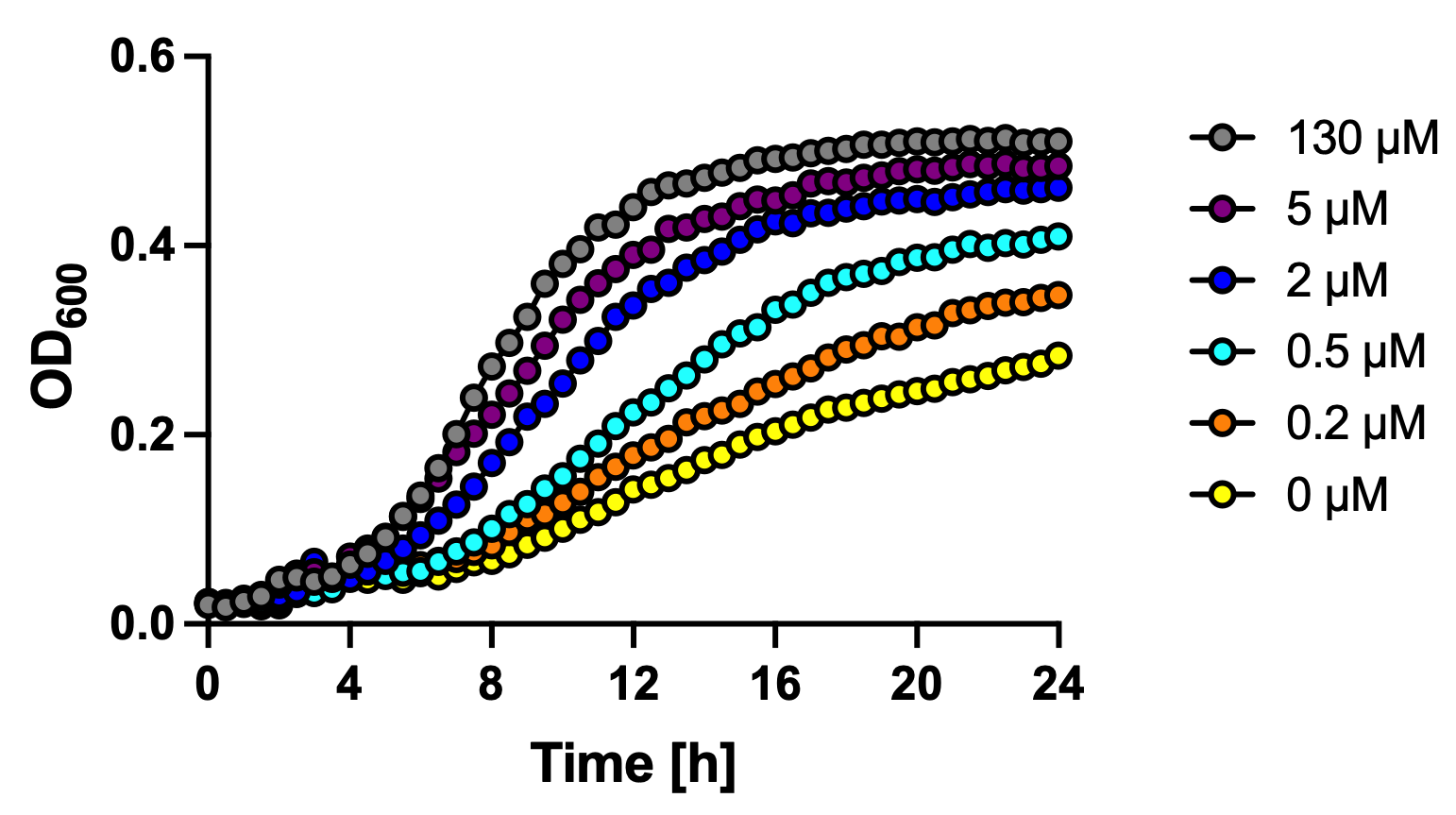
**

**S4 Fig: A reduction in histidine concentration in the medium leads to reduced growth of the *S. aureus* under acid stress conditions.** WT *S. aureus* LAC* containing the empty pCL55 (WT EV) was grown in CDM pH 4.3 medium with decreasing concentrations of histidine ranging from 130 μM to 0.2 μM or in the absence of histidine as indicated by the different symbols in the figure legend. The average OD_600_ readings from three experiments were plotted.
